# Maternal anaemia care in Kano state, Nigeria: an exploratory qualitative study of experiences of uptake and provision

**DOI:** 10.12688/f1000research.130980.3

**Published:** 2023-12-06

**Authors:** Aisha Kuliya-Gwarzo, Tara Tancred, Daniel Gordon, Imelda Bates, Joanna Raven

**Affiliations:** 1 Department of Haematology, Bayero University, Kano, P.M.B 3452, Nigeria; 2 International Public Health, Liverpool School of Tropical Medicine, Liverpool, L3 5QA, UK; 3 Physiotherapy, Brunel University, London, UB8 3PH, UK

**Keywords:** Maternal anaemia, experiences of care, care provision, qualitative research, Nigeria

## Abstract

**Background:**

Maternal anaemia (anaemia in pregnancy, childbirth, and the postpartum period) remains a persistent challenge, particularly in Kano State, Nigeria, which has the highest prevalence of maternal anaemia globally, at 72%.

**Methods:**

We conducted a qualitative study in Murtala Muhammad Specialist Hospital in Kano State, Nigeria. We aimed to identify factors constraining uptake and provision of maternal anaemia care, exploring perspectives across different stakeholders. We carried out 10 key informant interviews with policymakers and hospital managers, 28 in-depth interviews with healthcare providers and pregnant women using antenatal services and four focus group discussions with pregnant women’s husbands and mothers-in-law. Data were analysed thematically.

**Results:**

Issues with provision include a lack of provider training and guidelines specific to maternal anaemia and blood transfusion, insufficient staff to meet increasing demand, and inadequate resources. Issues with uptake include the inability to afford informal user fees, distrust in health services and the blood transfusion process, and a lack of understanding of the causes, consequences, and treatment for anaemia, resulting in poor uptake of care and adherence to treatment.

**Conclusions:**

This study recommends the implementation of standardized guidelines and training sessions to better support healthcare providers in offering quality services and increasing funding allocated to supporting maternal anaemia care. Education initiatives for service users and the public are also recommended to build public trust in health services and to improve understanding of maternal anaemia.

## Introduction

In Sub-Saharan Africa, approximately 57% of pregnant women experience anaemia. ^1^ This is problematic, as even mild anaemia increases perinatal mortality and early neonatal mortality, largely associated with preterm birth and intrauterine growth restriction. The odds of maternal mortality are also more than two times greater for severely anaemic women (haemoglobin < 7.0 g/dL), due to an increase in fatal postpartum haemorrhage. ^2^^–^^4^

The rate of prevalence of pregnancy-related anaemia in Nigeria is among the highest in the world, with prevalence ranging from 35% (Lagos) to 72% (Kano State). ^5^^,^^6^ Common causes of anaemia in pregnancy are iron and folate deficiency. ^2^ In Nigeria, parasitic infections such as malaria, hookworm and schistosomiasis, viral infections like human immunodeficiency virus (HIV), hepatitis B and C and inherited conditions affecting red blood cells such as sickle cell disease and thalassemia also contribute to anaemia. ^7^

Although physiological factors associated with anaemia have been well described, the health system and sociological factors are important. For example, the diagnosis and management of anaemia during antenatal care. Uptake of antenatal care in Nigeria is lacking, with only 67% of pregnant women attending antenatal care at least once, and only 56% attending four or more times. ^8^ Antenatal care quality may not be consistent, and in similar settings, the use of reliable approaches to measure anaemia occurs infrequently, and is often constrained by the availability of haemoglobin tests and reliance on symptom-based and clinical diagnosis. ^9^^,^^10^ Furthermore, though use of supplements or specific guidance around nutrition are key ways that anaemia can be managed in pregnancy, ^11^ adherence may be poor due to the gastrointestinal side effects of iron supplementation, lack of contact with local health services and misconceptions about anaemia. ^12^^,^^13^ An understanding of these factors is important to inform effective interventions for anaemia in pregnancy, which are culturally appropriate and address key deficiencies in healthcare provision for anaemia. ^14^ With the highest maternal anaemia prevalence in Nigeria, ^5^^,^^6^ Kano State is ideally situated to explore these issues.

This study aims to identify the factors that influence how maternal anaemia services are provided and used in Murtala Muhammad Specialist Hospital (MMSH) in Kano State. The findings are used to make practical and widely applicable recommendations to reduce anaemia in pregnant women within and beyond Nigeria.

## Methods

A qualitative phenomenological study was carried out to explore participant experiences around the provision and uptake of maternal anaemia care at MMSH.

### Study setting

MMSH is a secondary hospital in the Kano metropolis of Kano State in north-western Nigeria, with a population 13.4 million. Although there are more than 300 ethnic groups in Nigeria, most of the Kano population belong to the Hausa or Fulani ethnic groups, and are predominantly Muslim. ^15^ It is the largest government-owned hospital in Northern Nigeria and therefore draws a wide range of clients from across the state, making it a useful site to study our phenomenon of interest due to the diverse range of participants who attend this hospital. No fees are charged for consultation and admission, inclusive of antenatal services. This feature of service delivery also factored into our decision to carry out this study at this site, as it is more likely to bring in lower socioeconomic status families for whom anaemia is more likely to be a problem. There are 275 beds for obstetrics and gynaecology and a dedicated blood bank to collect from family members, which it screens for HIV and Hepatitis B and C using rapid testing.

### Sampling and data collection

*Key informant interviews*

Four key informant interviews were carried out with two policy makers and two hospital managers. Participants were sampled purposively due to their knowledge of policy-making that impacts care, interventions during pregnancy and childbirth, training of providers and oversight at hospitals. Prospective participants were contacted in-person or by phone by AKG and were given information about the study. They were given more than 24 hours to decide whether they wanted to participate or not. Interviews were conducted by AKG in a private room within the hospital or the participant’s workplace, wherever participants felt comfortable, and lasted for 30–100 minutes.

*In-depth interviews*

In-depth interviews were carried out with ten healthcare providers (doctors, nurses, laboratory staff, or community health extension workers who screen women and refer them to midwives) and 18 pregnant women. Providers were sampled purposively based on their experience in pregnancy anaemia management. Pregnant participants were sampled purposively to maximize diversity around the following characteristics: parity; duration of pregnancy; and prior experience of blood transfusion. Sampling was carried out until theoretical saturation had been reached.

AKG identified prospective healthcare providers through working with department heads, and approached them directly to provide information about the study. They were informed that this would be to gain their perspectives about providing anaemia care and would in no way be used to evaluate their performance. They had a week to consider their participation. Pregnant women were recruited in the waiting hall of the MMSH antenatal clinic. They were approached by AKG—who is in no way involved in their care—and were given information about the study, namely that they would be asked questions about their experiences of anaemia care and what might influence their uptake of this care. They had a full day to consider their participation.

Interview guides for providers focused on existing policies and services offered across the spectrum of care during pregnancy, childbirth and into the post-partum period, inclusive of the diagnosis and management of maternal anaemia. Interview guides for pregnant women focused on understanding of anaemia and experiences of care during pregnancy and childbirth. These guides were developed following an extensive reading of the literature and author AKG’s intimate knowledge of the context.

Interviews with providers and pregnant women were scheduled at their convenience, and for providers, took place in private spaces in the hospital. Some women needed permission from husbands or relatives and were given a contact number to confirm participation. Most interviews with pregnant women were held in the matron’s office at the antenatal clinic. However, those who confirmed participation after obtaining permission had their interviews conducted at AKG’s office in Aminu Kano Teaching hospital. Interviews took 30–40 minutes.

*Focus group discussions*

Four focus group discussions (FGDs) were held with women’s relatives, each with six participants: two groups of six husbands and two groups of six mothers-in-law. Snowball sampling was used to identify participants, as women who participated in interviews were asked to inform their husbands and their mothers-in-law about the research and to invite them to participate in FGDs. Those who agreed were contacted by telephone and FGDs were organized.

Discussion guides focused on participants’ understanding of anaemia and perceptions of care received by their partners or daughters-in-law during pregnancy and childbirth.

All interviews and FGDs were carried out by AKG—who has extensive training in social science methodology—in Hausa or English. All interviews and FGDs were audio-recorded, transcribed verbatim, and later translated into English if necessary. Handwritten notes to capture non-verbal communication were made during interviews and FGDs by a research assistant. Interviews took 20–60 minutes and FGDs took 45–70 minutes.

### Data analysis

Thematic analysis was used. ^16^ All steps of the data analysis were carried out by AKG, TT, and DG. Data were read and re-read for familiarity. Line-by-line inductive coding was carried out and a coding framework was established in Excel. Codes were grouped into higher-order codes and themes. Applying and refining the coding framework was repeated until no new codes were generated and thematic saturation was reached, at which point, participant recruitment ceased.

The following four themes emerged: guidelines and policies for managing anaemia; quality of care; resources and financing; and knowledge, attitudes and practices of women and family members. These are described in the results that follow.

## Results

### Participant characteristics

All participants are described in Table 1.

**Table 1.** Participant characteristics.

	Participants/role	Age	Sex	Level of education/certification	Total participants	
**Key informant interviews**	**Policy makers**	43	M	Medical qualification	2	4
		56	M	Medical qualification		
	**Hospital managers**	45	M	Medical qualification	2	
		50	M	Medical qualification		
**In-depth interviews**	**Healthcare providers**	60	F	Certificate as nurse/midwife	10	
		45	F	Certificate as nurse/midwife		
		49	F	Certificate as nurse/midwife		
		35	M	Medical qualification		
		32	M	Medical qualification		
		45	M	Medical laboratory science degree		
		30	F	Certificate as nurse/midwife		
		48	F	Certificate as nurse/midwife		
		30	F	Diploma (Community health extension worker)		
		39	F	Diploma (Community health extension worker)		

	Participant	Age	Parity	Level of education	Total participants	
**In-depth interviews**	**Pregnant women**	30	5	Secondary school	18	
		29	4	Primary school		
		20	2	Secondary school		
		25	1	Secondary school		
		19	1	No education		
		26	4	No education		
		38	8	No education		
		20	0	No education		
		35	2	No education		
		44	5	No education		
		26	3	Secondary school		
		24	4	No education		
		40	7	No education		
		33	5	No education		
		40	11	No education		
		20	3	Secondary school		
		22	3	No education		
		19	1	Secondary school		

	Participants	Age	Number of children	Level of education	Total participants	
**Focus group discussions**	**Mothers-in-law (Focus group discussions 1 and 2)**	45	8	Primary school	12	
		51	2	No education		
		54	7	No education		
		39	5	Primary school		
		46	3	Primary school		
		59	9	No education		
		30	7	Primary school		
		50	10	No education		
		60	5	No education		
		43	3	No education		
		42	6	Primary school		
		55	8	No education		
	**Husbands (Focus group discussions 3 and 4)**	46	2	Diploma	12	
		35	4	Secondary school		
		52	3	Primary school		
		30	3	College certificate		
		38	5	Primary school		
		35	6	Secondary school		
		36	2	Diploma		
		37	4	Secondary school		
		30	3	Diploma		
		30	3	College certificate		
		38	5	Primary school		
		35	6	Secondary school		
Total all participants					56	

### Guidelines and policies for managing anaemia are available, but not always trained to users or implemented

Key informants and providers summarized the guidelines and policies for managing anaemia. Pregnant women are seen first between 20 and 28 weeks. Before consultation, they receive anaemia education on topics including: issues in pregnancy, childbirth and when breastfeeding; risk factors; signs and symptoms; prevention using diet; treatment; and the importance of child spacing. During antenatal care consultations, pregnancy is confirmed using last menstrual period, the estimated due date is calculated, and the women are tested for blood group, packed cell volume and infections.

*‘We offer free laboratory tests at the first antenatal visit which include [packed cell volume] check, blood group and HIV screening and urinalysis. They are all free.’* (Hospital manager)

Healthcare professionals are expected to treat anaemia based on an individual assessment using their clinical judgement, the client’s estimated packed cell volume, severity of anaemia and likely cause. Most pregnant women are administered precautionary oral iron supplements because iron deficiency anaemia is very common. Women with severe anaemia are thought to be from rural areas and may not have attended antenatal care. Blood transfusion is indicated in those with associated dizziness and weakness.

Providers reported there was no concerted effort to ensure that staff were trained on guidelines for anaemia diagnosis and management, or that guidance was fully implemented or monitored.

*‘A deliberate policy or methodology to distribute most of these guidelines is usually not in place.’* (Policymaker)

Healthcare professionals reported that, as per Kano state policy, care to women during pregnancy, childbirth, and in the postpartum period is free, including a packed cell volume screen, supplements, and medication to prevent/treat anaemia and dietary advice. Participants clarified that blood transfusions were also included for free, provided patients’ relatives donated blood to replace units used.

*‘We offer blood transfusion which is also free […] even the donors that will come and donate the blood they will all be screened free.’* (Hospital manager)

*‘The patient has to bring a donor […] we give the transfusion free but the only thing is that she needs to bring the donor.’* (Policymaker)

As subsequent results suggest, there is a mismatch between the policies and guidelines that exist and the practices that take place.

### Quality of anaemia care is constrained

Healthcare providers were knowledgeable about maternal anaemia and its often-complex origins. There was recognition that there is not always the ability to address all possible causes, which would necessitate nutrition counselling, iron, and folic acid supplementation—and adherence, malaria prophylaxis, deworming, and child spacing, among other interventions. The nutritional and child-spacing aspects were understood as being culturally mediated and difficult to change.

*‘Anaemia associated with pregnancy has a lot of issues—it is more like multifactorial. If you look at the way our women are, repeated pregnancies and deliveries actually leads to loss of blood at every delivery, or rather, at every labour, and so many of them, before even the labour, they may have a miscarriage and this also causes a lot of bleeding … The other issue is that if you look at it, our cultural habit of eating … our eating habits do not contain many of the green vegetables that may have the haemoglobin, or rather, the iron component that should be consumed daily. The other issue is … malaria … another key role in developing anaemia [is] haemorrhage, so there are so many things and eh, I must say, anaemia is playing a very bad role in the issue of pregnancy, in pre-term delivery and other issues like that.’* (Healthcare provider)

Healthcare professionals and women reported several issues that affected access to and the quality of care: high staff turnover and inadequate staffing, particularly of specialist physicians; high demand for care; insufficient remuneration for staff; and unsatisfactory supervisory support.

*‘The gap is wide because we have a lot of patients here and the staff are few … let’s take the eclamptic patients. We have almost 20-something beds in the eclamptic ward, but you will see only one or two staff running the shift. At times we have a patient that is coming to theatre, and for God’s sake, if it is only one staff, who will take care of the ward and who will come to the theatre, do you understand?’* (Healthcare provider)

*‘We have a kind of brain drain if so to say or people are leaving the service of the state for a greener pasture, because if you compare the package of state and federal institutions, definitely there is a sharp difference. So, really, there is difference from the state to the federal institution, so most of our staff leave for the federal institutions and this may account for why staff are not enough in this facility or in the state generally.’* (Healthcare manager)

Hospital managers, women and relatives reported that free provision was responsible for a drastic increase in women attending the service, which placed a strain on service delivery. This is because the service was attracting users from nearby states such as Jigawa, as well as an influx of clients previously unable to afford care.

*‘So, like Kano, everybody is trooping into Kano [to access maternal care]. So, if you plan for like 10 million people, then you end up having 15 million for example.’* (Policymaker)

Healthcare professionals reported that staff often did not take time to properly consult with patients, in addition to huge workload, because of other commitments outside the hospital.

*‘Some are usually in a hurry to finish and go for other issues of theirs. Either some of them may want to go for private practice, or somewhere they have a personal issue.’* (Healthcare provider)

Consequently, time for one-on-one counselling regarding medication compliance was limited, and there was reduced ability to diagnose women with anaemia in the early stages. Many women and relatives felt unable to discuss problems with staff because there was a lack of privacy during consultations due to crowded waiting areas. Staff also appeared rushed and disengaged, which women regularly noted prevented them from raising potential concerns.

*‘The time you get is very short and it takes long before you see them because there are too many people to be seen and there is no privacy.’* (Pregnant woman)

*‘[After describing a complication and being asked why the nurse was not informed:] I couldn’t talk to her, she seemed to be in a hurry and there were many people in the room waiting.’* (Pregnant woman)

Appropriate maternal anaemia care may be constrained by a lack of training and, as above, lack of specific guidelines. For example, providers understood that maternal anaemia is defined as a low packed cell volume in pregnancy or the postnatal period and relied on estimates of this to diagnose anaemia. However, providers’ perceptions of the cut-off values at which anaemia would be indicated—some stated 20%, others 25% and others 30%—were inconsistent.

*‘Investigations and diagnosis is by [packed cell volume] only … honestly, we consider cost most of the time. We think if we ask for a full blood count it may be too expensive, so the only thing we used to ask for is [packed cell volume].’* (Healthcare provider)

For blood transfusions, staff relied on knowledge from books and the experience of senior colleagues as opposed to local standards of practice, which were not used. Hospital managers and healthcare providers reported no formally scheduled staff training on maternal anaemia, with staff required to use their experience to provide on-the-job training.

*‘We train [new staff] here [in maternal anaemia care] as they come, on the job. But no formal training.’* (Hospital manager)

### Resources and financing for anaemia care are inadequate

Healthcare providers reported that they were often unable to diagnose causes of maternal anaemia because specific tests were unavailable or too costly. As a result, reliance was on clinical and symptom-based diagnosis, which can often only identify anaemia when it is very severe.

*‘We think if we ask for full blood count it may be too expensive so the only thing we use to ask for is [packed cell volume].’* (Healthcare provider)

*‘Diagnosis of anaemia, usually the patient will be complaining of dizziness or palpitation. When they come, we have to interview them, we will do physical examination from head to toe, then their conjunctiva.’* (Healthcare provider)

Furthermore, causes of anaemia were rarely investigated, due to too much perceived demand, which may constrain appropriate care.

*‘I don’t know of my colleagues but I know I don’t always … investigate the cause [of anaemia]. I know not many people used to be very keen on diagnosing the cause, we just assess if she needs transfusion, then we transfuse, if she doesn’t need transfusion, because maybe we considered the population is too much or something. We are not used to really investigating the cause.’* (Healthcare provider)

Healthcare providers reported stocks of medication were often inadequate because demand out-weighed supply.

*‘This program has not been effective and efficient and they will only give drugs that will last for 2 weeks for the whole quarter.’* (Hospital manager)

Hospital managers also acknowledged that reporting stockouts was not always well-received at higher levels. These participants therefore reported attempts to generate resources and support free care by diverting funds away from the drug revolving fund—a fund generated by drug sales, laboratory tests and other hospital services intended to maintain services.

*‘You don’t ever say that the drug is out of stock because […] if the government or any official that is close to the government gets to know of this […] they will remove you.’* (Hospital manager)

*‘We take some funds from the [drug revolving fund] and give it to this free maternity but […] in a way we are decapitalizing the [drug revolving fund].’* (Hospital manager)

Despite existing policies, women and relatives reported often purchasing medications from pharmacies—or from staff—along with making other out-of-pocket expenses, like paying for packed cell volume testing. Women and their husbands corroborated this finding, widely reporting the expenses associated with seeking care. These expenses were seen as making uptake of care, or use of appropriate medications, inaccessible for some due to widespread poverty.

*‘If we are out of stock, we ask them to buy. If they can afford to buy, fine, if they cannot afford to buy then may God save them.’* (Hospital manager)

*‘There is no free service, it is just politics […] Everything needed for delivery ranging from hand gloves, razor blades and others, have to be provided by the family.’* (Husband)

Blood availability remains a major constraining factor in treating anaemia. In emergencies, medical staff collecting blood for transfusion from the blood bank are responsible for ensuring that, prior to patient discharge, the patient’s family provide replacement blood.

*‘They said they will never agree to do that [give blood without a replacement donation] because some of the doctors do not insist that the patient replaces the blood.’* (Healthcare provider)

Because blood type may be rare and consumables such as blood bags must be bought, significant delays can occur. Relatives are often not available when there is an urgent need, so it can take hours to obtain a donation, screen for infections and crossmatch. The most common reason for rejection of donated blood is the presence of Hepatitis B virus markers. Despite need for transfusion, if blood, or a specialist able to administer the transfusion, is not available, it will not take place. Multiple participants spoke of instances where they or their partner were awaiting a transfusion that never took place.

*‘Well the person to donate came after a few hours and donated but in the end she was not even transfused.’* (Husband)

Participants reported that obtaining donor blood is slow and difficult due to: negative cultural connotations of having another person’s blood in one’s system; low level of awareness of maternal anaemia in the public; fear of being diagnosed with an unknown disease; belief that they do not have enough blood to donate; belief that a financial burden will be incurred; and public distrust of transfusion services. The difficulty in sourcing donors leads some to abscond from the ward after transfusion without replacing blood. Consequently, blood bank staff often refuse to accept emergency requests for blood, exacerbating the issue.

*‘It means I have a different blood of someone I don’t know of his character and he may not be a good person.’* (Pregnant woman)

*‘We were told to provide the donors and it was quite distressing since she had [need for] transfusion and not many people were willing [to donate blood]. They are usually scared of giving blood … maybe they think you can contact disease from that and they sometimes say they do not have enough to give. Some are also scared of testing [for infections]. You know people know the blood will be tested.’* (Husband)

### Knowledge, attitudes and practices of women and family members may constrain uptake of care and adherence to prescriptions

Most women and relatives described anaemia in terms of its symptoms, some mistakenly equating anaemia to blood pressure. Most reported poor diet and blood loss as causes, but inconsistently related repeated pregnancies at short intervals to anaemia. Relatives mostly related maternal anaemia to low blood levels from inadequate diet or early pregnancy. Healthcare professionals reported that often, even after education, women still become anaemic. There are several reasons for this. Some women reported that many did not engage fully with education and were distracted, while others reported that the time would have been better used for one-on-one consultations.

*‘To those that listen it is very useful but many do not listen […] some of the women would rather listen to their phone radios.’* (Pregnant woman)

Healthcare professionals reported that medication and dietary advice are sometimes not followed, in part due to lack of understanding, inconsistent antenatal care attendance, illiteracy and lack of anaemia education as well as poor perception of hospital care. Prescriptions are often lost and not re-administered until the following visit.

*‘Honestly, some don’t take their prescriptions, some are negligent. When the drugs are prescribed, they don’t take it. Like during antenatal, there are 4 visits, some will come for 3 visits, but they will not even know what drugs they have been prescribed. And we issue health talks every day, [pregnant women] are being told the importance of taking their drugs, medical tests, but some will tell you they don’t know what they have been asked to do.’* (Healthcare provider)

*‘[Pregnant women in the community] will say they are being harassed … some will not agree, you see, this hospital, rumours have been circulating saying people insult each other, the doctor insults this and that. Some they prefer to see male doctors than female doctors … because the male doctors don’t harass you. If you are sick in this hospital, male doctors will look after you better, they follow standard procedures bit by bit, they ask you this and that, but female doctors will not mind … some will not even listen to you; they will harass and embarrass you.’* (Pregnant woman)

Some women dislike taking the medications like iron and folic acid supplementation, which are prescribed routinely, while others were not aware that drugs were prescribed and were unclear on medication guidance.

*‘Yes, there are some that tell you when they take the drugs they will vomit [because] they don’t like it.’* (Healthcare provider)

Many women continued to believe that there was no cure for maternal anaemia, but that a range of traditional herbal treatments alleviate symptoms. Traditional healers are cheap, known to the family, live locally, can provide care to women at home, and women do not require permission to seek their assistance.

*‘They will start taking the traditional concoction to give the woman [in the village]. Those are the women that will come in with severe anaemia and cardiac failure, but this is rare.’* (Hospital manager)

*‘Traditional health care is good because is at home you can help yourself with what you can afford.’* (Pregnant woman)

However, women and relatives explained that if traditional treatments fail, the condition may become severe, and women will eventually have to seek hospital care.

*‘They believe in the [traditional birth attendants] so much. They only come to hospital when it is late and the woman would have suffered enough.’* (Husband)

However, attending hospital care was sometimes seen as burdensome, taking women away from domestic responsibilities, which must be completed upon their return.

*‘It is like everybody is in a hurry and sometimes the women would rather listen to their phone radios … some want to go back home early—maybe they have not finished their chores. Some will be chatting and maybe some would like to visit the market.’* (Pregnant woman)

Women, husbands, and relatives saw anaemia as a source of familial friction, especially because women ordinarily needed to gain the permission of husbands and/or mothers-in-law to attend hospital appointments. Though most husbands reported supporting their wives for healthcare costs, healthcare professionals reported that women were often financially dependent on husbands who could not, or would not, provide enough money for women to treat their anaemia and effectively feed the family. Poor economic status of women often disempowers them and further constrains their decision-making.

*‘If you prescribe the drugs the husband will try to get the cheaper alternative which may not be good.’* (Pregnant woman)

*‘If you look at cultural issues, our family setting where you have repeated pregnancies on and on, you see a woman having 8, 10 deliveries and her economic status is very poor … there is no empowerment, women are totally under-powered and they are fully dependent, 100% dependent on … what is being dumped on them. If [her husband] has only N200, then he will say, ‘ok take this N200, know how to manage it’, that will be the morning fee for breakfast for her and probably with about 5 children, so who will eat? She’d rather give it to the children and starve herself up to the time when food is available, and if the food is available, is not nutritious in the sense that probably, carbohydrate is more than the protein you can get and then usually they lack the elements that can be used by the body to produce the blood, like the iron.’* (Healthcare provider)

Relatives reported that women did not always accurately relay information to their husbands from the clinic because they may have forgotten or misunderstood. They reported that women were sometimes too shy to raise problems with staff. Some women added that being attended to by male staff would aggravate some husbands.

*‘They don’t like a man to attend to their wife because of jealousy.’* (Pregnant woman)

One woman explained that blood transfusions could also cause arguments between husbands and relatives, because they feel that the need for a transfusion means that the husband does not take proper care of his wife.

*‘[Blood transfusion] results in conflicts […]. The general feeling is that the husband has failed.’* (Husband)

Most women reported that they would be happy to have their husbands in attendance at antenatal appointments, because they felt their husbands could communicate concerns directly to caregivers and were more likely to believe advice directly from hospital staff. Women reported that most husbands did not attend because of cultural norms, particularly the fear of embarrassment if seen accompanying a woman to the antenatal care or for childbirth. Some husbands responded that they would be glad to attend the antenatal care with their wives, but that this would mean taking time off work, being in a crowded space in the clinic and interacting with other women. Consequently, only a few reported attending and instead supported their wives financially.

*‘The problem with this [information given in antenatal care] is that we are all women. Most of us do not earn and we depend on our husbands, and since the husband is not there with you, even if you tell him, he may think you are just saying it because you want to eat this and that, but if he is there and hears what is being said from the nurses, he will believe it more.’* (Pregnant woman)

*‘Financial is the most important support we can give, and outside that, it is to wish them safe delivery.’* (Husband)

## Discussion

Key findings from our study highlight that, in Kano, key issues with provision of care for maternal anaemia centred on the service being under-staffed and under-resourced to deal with demand. Tests to determine the causes of maternal anaemia were sometimes unavailable. Providing blood for transfusions in the event of severe maternal anaemia was also problematic, due to difficulties ensuring the replacement of blood. Healthcare providers have limited time and space to provide comprehensive education about maternal anaemia and how it is treated, leaving some misconceptions around treatment unchecked. Uptake of maternal anaemia care was constrained by the understanding that maternity services are not free in practice. Women’s reliance on their husbands for financial resources and permission to seek care was a barrier. Husbands were rarely involved in antenatal care due to social norms, embarrassment, and practical constraints around availability of space in the hospital.

These issues in provision and uptake suggest that anaemia is not being fully addressed in this vulnerable population, contributing to unacceptably high levels of anaemia and peripartum mortality seen in Kano state. ^8^ Findings around poor quality of maternal anaemia care are consistent across other Nigerian settings and low- and middle-income countries (LMICs). ^17^^–^^19^ In Nigeria—and LMICs with “free” maternal care policies—the expectation of both indirect or direct payments for maternity services limits uptake of care and pushes women to receive informal care from traditional healers. ^20^^–^^24^ Anaemia is complex, and while our results highlight a generally good understanding of anaemia as a consequence of inadequate nutrition or blood loss among participants who knew what anaemia was, the role of child spacing to prevent anaemia was not well-understood. There is likely to be misunderstanding of how anaemia can be prevented at a household level, which is illustrated through the very high prevalence of maternal anaemia (72%) in Kano. ^6^^,^^7^ These findings are consistent with other settings, in which prevention of anaemia at the community level, particularly through nutrition, was poorly understood. ^25^^–^^28^ As has been found in other LMIC settings, community-level education around maternal anaemia or initiatives to support community-level distribution of iron and folic acid supplements—possibly mediated through community health extension workers—may increase adherence to medical guidance, improve buy-in from husbands and other family members and equip families to make more informed healthcare decisions. ^29^^–^^32^ Targeted, culturally sensitive community education, especially involving young adults, about the purpose and value of blood donation may also reduce stigma and misconceptions around blood donation and increase the number of repeat, voluntary blood donors. ^33^^–^^35^ Further research is needed to identify the most effective ways of providing such education, and how this can be done to promote preventative measures for those who are not yet involved with the service.

Many of the issues with care provision are a consequence of inadequate funding, an issue that resonates across maternal health services more broadly. ^36^ Interventions to improve supply chain management for resources like haemoglobin tests have been successful elsewhere in improving diagnosis of maternal anaemia. ^37^ Further research is required to identify other sources of funding for maternal anaemia services, or ways in which the system can be restructured to more efficiently use funds.

Given poor adherence to oral iron supplementation, as was found in our study and many others, intravenous iron infusions may be a promising alternative to treat maternal anaemia. Like blood transfusion, these provide an immediate increase in haemoglobin and are generally well-tolerated. ^38^ Although this was not present in our study site at the time of data collection, more recent research in Nigeria, including in Kano state, is ongoing to specifically assess its utility in treating anaemia in pregnant women. ^39^

For blood transfusion, ^40^ standards of care for maternal anaemia are present, but inadequately implemented. The enforcement among maternity staff of standardized guidelines and practice for anaemia diagnosis and treatment would be important. Quality improvement approaches such as standards-based audit may be helpful in this respect. ^41^ Furthermore, the introduction of regular training on issues such as patient communication, maternal nutrition and anaemia-related healthcare may lead to a higher standard of service. ^42^^–^^44^

Overall, it is clear that, though anaemia in pregnancy is well understood from a physiological standpoint, with a significant evidence-base around its prevention, management, and treatment, it remains highly prevalent. ^45^^,^^46^ There is a gap around perspectives regarding provision or receipt of care for maternal anaemia, which is needed to understand where improvements in service delivery or community sensitization could be made to drive improved evidence-based practice, adherence to treatments, and uptake of care. ^47^^,^^48^

### Strengths and limitations of the study

A strength of this research is that it draws on multiple perspectives from a range of health systems actors, which allowed both supply- and demand-side factors to be highlighted. Further, there are limited studies around experiences of maternal care services specific to anaemia, which is a significant problem globally, but especially in Sub-Saharan Africa.

A key limitation is that interviews were conducted with women who were already accessing services. These participants had overcome many barriers to accessing these services, and there was an increased likelihood that those interviewed were more sympathetic to institutional healthcare. It is possible that those who were unable to access these services experience other barriers that are not discussed here, requiring further research.

## Conclusions

This study explored maternal anaemia services use and provision at MMSH, by drawing from diverse perspectives across a variety of health systems stakeholders. Issues with care provision included a lack of support for healthcare professionals and an under-resourced service attempting to meet increasing demand and difficulties in sourcing donor blood. Issues with care uptake were hidden costs, women’s dependence on husbands for finances and permission to seek care and a lack of anaemia literacy. Understanding these barriers may inform future interventions for anaemia in Kano and help to improve services. Many of these findings may be generalizable to other parts of Nigeria when providing care for maternal anaemia. The importance of better resourced facilities and standardized training and guidelines is likely to be cross-cutting, applying to other maternity services as well. Supporting the uptake of maternal anaemia services is critical, and community education is a low-resource way of improving such uptake.

## Ethics and consent

Ethical clearance was obtained from the research ethics committees of the Liverpool School of Tropical Medicine (20 ^th^ July 2016, Reference: 16-015) and Aminu Kano Teaching Hospital (19 ^th^ June 2016). We confirm that this research was conducted fully in accordance with the ethical principles for carrying out medical research with human participants as stipulated in the Declaration of Helsinki.

All participants provided written informed consent after receiving detailed information about the study. For illiterate participants, information was read to them, checked for understanding and a literate witness signed on their behalf. All data were collected in private spaces to ensure privacy and confidentiality, were anonymized, and identifying details were removed. Participants were given a unique identified. All data were uploaded and stored digitally. All files were encrypted and password protected to ensure confidentiality.

## Data Availability

Data are not deposited in an open access repository as participants were not asked to consent to this. Though all data are anonymized, key informants occupy specific roles, and there may be privacy concerns around their data if reviewed by someone very familiar with the context. However, anonymized data sets intended to support secondary data analysis, for example, within a systematic review or qualitative meta-synthesis, may be made available upon reasonable request to the corresponding author, AKG (at akgwarzo.hae@buk.edu.ng or aisha.kuliya@gmail.com).
