# Supplementary material for: Maternal anaemia care in Kano state, Nigeria: an exploratory qualitative study of experiences of uptake and provision
Source: F1000Res. 2023 Dec 6;12:288. Originally published 2023 Mar 15. [Version 3] doi: 10.12688/f1000research.130980.3 (PMC10905168; doi:10.12688/f1000research.130980.3)
Supplement: Peer review history

# Reviewer report: Reviewer response for version 3

**DOI:** 10.5256/f1000research.159666.r228235

**Published:** 2024-02-09

**Authors:** Apurva Kumar Pandya

**Affiliations:** 1 Department of Health Policy, Management and Behaviour Science, Indian Institute of Public Health, Gandhinagar, Gujarat, India

The authors have addressed all comments. A manuscript can now be approved.

Is the work clearly and accurately presented and does it cite the current literature?

Partly

If applicable, is the statistical analysis and its interpretation appropriate?

Yes

Are all the source data underlying the results available to ensure full reproducibility?

Yes

Is the study design appropriate and is the work technically sound?

Yes

Are the conclusions drawn adequately supported by the results?

Partly

Are sufficient details of methods and analysis provided to allow replication by others?

Partly

Reviewer Expertise:

health promotion, health communication, behaviour change communication, health technology assessment, qualitative research, and health system and policy research

I confirm that I have read this submission and believe that I have an appropriate level of expertise to confirm that it is of an acceptable scientific standard.

# Reviewer report: Reviewer response for version 3

**DOI:** 10.5256/f1000research.159666.r228236

**Published:** 2023-12-16

**Authors:** Amy Brenner

**Affiliations:** 1 London School of Hygiene and Tropical Medicine, London, UK

Thank you for incorporating the paragraph suggested in the discussion.

Is the work clearly and accurately presented and does it cite the current literature?

Yes

If applicable, is the statistical analysis and its interpretation appropriate?

Not applicable

Are all the source data underlying the results available to ensure full reproducibility?

Partly

Is the study design appropriate and is the work technically sound?

Yes

Are the conclusions drawn adequately supported by the results?

Yes

Are sufficient details of methods and analysis provided to allow replication by others?

Yes

Reviewer Expertise:

Maternal health, postpartum haemorrhage, traumatic haemorrhage, randomised trials, anaemia

I confirm that I have read this submission and believe that I have an appropriate level of expertise to confirm that it is of an acceptable scientific standard.

# Reviewer report: Reviewer response for version 2

**DOI:** 10.5256/f1000research.158290.r219995

**Published:** 2023-11-27

**Authors:** Amy Brenner

**Affiliations:** 1 London School of Hygiene and Tropical Medicine, London, UK

I cannot see any mention of IV iron as they suggest in the changes. Please can you amend the statement on what has been changed or can they actually do as they said? Happy to approve the article with either but at the moment the current version doesn’t reflect what the authors say in their response.

Is the work clearly and accurately presented and does it cite the current literature?

Yes

If applicable, is the statistical analysis and its interpretation appropriate?

Not applicable

Are all the source data underlying the results available to ensure full reproducibility?

Partly

Is the study design appropriate and is the work technically sound?

Yes

Are the conclusions drawn adequately supported by the results?

Yes

Are sufficient details of methods and analysis provided to allow replication by others?

Yes

Reviewer Expertise:

Maternal health, postpartum haemorrhage, traumatic haemorrhage, randomised trials, anaemia

I confirm that I have read this submission and believe that I have an appropriate level of expertise to confirm that it is of an acceptable scientific standard.

# Response

**Published:** 2023-11-27

**Authors:** Tara Tancred

**Affiliations:** Liverpool School of Tropical Medicine, UK

Thank you for your re-review and approval. 

 We added in the following paragraph with references, so will follow up with the editorial team about why this was not reflected in this revised version. 

 Given poor adherence to oral iron supplementation, as was found in our study and many others, intravenous iron infusions may be a promising alternative to treat maternal anaemia. Like blood transfusion, these provide an immediate increase in haemoglobin and are generally well-tolerated. [TT1] Although this was not present in our study site at the time of data collection, more recent research in Nigeria, including in Kano state, is ongoing to specifically assess its utility in treating anaemia in pregnant women. [TT2] 

[TT1]Please add this reference here: Bonovas S, Fiorino G, Allocca M, Lytras T, Tsantes A, Peyrin-Biroulet L, et al. Intravenous versus oral iron for the treatment of anemia in inflammatory bowel disease: a systematic review and meta-analysis of randomized controlled trials. Medicine. 2016;95(2):e2308.

[TT2]Please add this reference here: Afolabi, B.B., Babah, O.A., Akinajo, O.R. et al. Intravenous versus oral iron for iron deficiency anaemia in pregnant Nigerian women (IVON): study protocol for a randomised hybrid effectiveness-implementation trial. Trials 23, 763 (2022). https://doi.org/10.1186/s13063-022-06690-2

# Reviewer report: Reviewer response for version 1

**DOI:** 10.5256/f1000research.143782.r205949

**Published:** 2023-10-17

**Authors:** Amy Brenner

**Affiliations:** 1 London School of Hygiene and Tropical Medicine, London, UK

Thank you for the opportunity to review this article about the provision and uptake of services for maternal anaemia diagnosis/treatment in Kano state, Nigeria. Firstly I would like to thank the authors for highlighting this important public health issue, which is a major risk factor for postpartum haemorrhage (PPH).

 You may be interested in a recent analysis that explored the association between haemoglobin level, PPH and death or near miss in over 10,000 moderately and severely anaemic women, including women in Nigeria recruited into the WOMAN-2 trial ( https://www.sciencedirect.com/science/article/pii/S2214109X23002450). 

 An important cause of anaemia in women of reproductive age that is often overlooked (and has been overlooked by the author) in the prevention and treatment of anaemia is menstrual bleeding, particularly heavy menstrual bleeding. The authors may wish to comment on this. e.g. https://doi.org/10.1016/S0140-6736(20)32718-5.

 The article reports some very interesting insights from FGDs and interviews with HCPs, pregnant women and relatives. It highlights gaps in health services/resources/guidelines/policies/awareness/education, which may be targeted to improve the diagnosis and treatment of anaemia in pregnant women before they give birth. The discussion highlights well some possible actions to overcome the complex barriers to care for maternal anaemia.

 The author says that healthcare providers were knowledgable about maternal anaemia but this is to be expected as the sample was purposively selected 'based on their knowledge and experience in pregnancy anaemia management'. This statement should be removed from the results to avoid the reader misconstruing this as evidence that HCP training on maternal anaemia is sufficient.

 It is interesting that intravenous iron infusion is not mentioned as a treatment for maternal anaemia. The authors should comment on this in the discussion. I believe there is an ongoing study of IV iron being conducted in Lagos...

Is the work clearly and accurately presented and does it cite the current literature?

Yes

If applicable, is the statistical analysis and its interpretation appropriate?

Not applicable

Are all the source data underlying the results available to ensure full reproducibility?

Partly

Is the study design appropriate and is the work technically sound?

Yes

Are the conclusions drawn adequately supported by the results?

Yes

Are sufficient details of methods and analysis provided to allow replication by others?

Yes

Reviewer Expertise:

Maternal health, postpartum haemorrhage, traumatic haemorrhage, randomised trials, anaemia

I confirm that I have read this submission and believe that I have an appropriate level of expertise to confirm that it is of an acceptable scientific standard, however I have significant reservations, as outlined above.

## References

[1] : Maternal anaemia and the risk of postpartum haemorrhage: a cohort analysis of data from the WOMAN-2 trial. *Lancet Glob Health* .2023;11(8) : 10.1016/S2214-109X(23)00245-0 e1249-e1259 10.1016/S2214-109X(23)00245-0 37390833 PMC10353972

# Response

**Published:** 2023-10-29

**Authors:** Tara Tancred

**Affiliations:** Liverpool School of Tropical Medicine, UK

Many thanks to the reviewer for their review of our manuscript. We have responded inline in bold to the queries raised. 

 An important cause of anaemia in women of reproductive age that is often overlooked (and has been overlooked by the author) in the prevention and treatment of anaemia is menstrual bleeding, particularly heavy menstrual bleeding. The authors may wish to comment on this. e.g. https://doi.org/10.1016/S0140-6736(20)32718-5.

***We absolutely agree this is an important contributor to anaemia, though not necessarily maternal anaemia, as our participants are pregnant (and therefore not menstruating).***

 The author says that healthcare providers were knowledgeable about maternal anaemia but this is to be expected as the sample was purposively selected 'based on their knowledge and experience in pregnancy anaemia management'. This statement should be removed from the results to avoid the reader misconstruing this as evidence that HCP training on maternal anaemia is sufficient.

***Thank you for this comment—to be clear, we did not intentionally select those healthcare providers who demonstrated high levels of knowledge—simply those with the responsibility and experience of providing anaemia care (through which knowledge would be expected). We have clarified this in the text.***

 It is interesting that intravenous iron infusion is not mentioned as a treatment for maternal anaemia. The authors should comment on this in the discussion. I believe there is an ongoing study of IV iron being conducted in Lagos...

***Thank you for the recommendation. This is not used in the study site, but we have briefly commented on this in the discussion.***

# Reviewer report: Reviewer response for version 1

**DOI:** 10.5256/f1000research.143782.r201662

**Published:** 2023-09-26

**Authors:** Apurva Kumar Pandya

**Affiliations:** 1 Department of Health Policy, Management and Behaviour Science, Indian Institute of Public Health, Gandhinagar, Gujarat, India

Authors have chosen important topic. However, there are some areas that require authors' attention to improve scientific rigour. Suggestions for improvements are as follow:

 Introduction

- As majority participants are from Hausa or Fulani ethnic group, there should be brief paragraph on ethic groups in Nigeria.

Methodology: 

 Authors should re-organize this section as follows:

- Research design: Specify which qualitative research design was used.
- Study setting: provide justification why this setting was selected.
- Explain some characteristics of this ethic group. Hausa or Fulani ethnic groups 
- Sampling technique should be explicitly mentioned.
- Research tools: Explain how each research tools were developed.
- Data collection procedure: Explain who and how data was collected? In which language data was collected? Was data collector trained in qualitative interviews/qualitative research? What was the duration of data collection? 

Ethical considerations: Explain how authors ensured human rights protection of participants. Explain how authors dealt with ethical dilemma if they had encountered.

 How qualitative data managed need to be explained (deidentification, storage of data, protection of privacy, etc.)

 Data analysis: Analytic framework used by authors should be explained with appropriate reference (citation) and steps followed in analyzing qualitative data.

 Results: This section is poor. It looks superficial. Authors claim inductive coding was used; however, results do not reflect that.  It lacks triangulation of data from different sources and analytic rigour. Authors should follow thematic analytic framework (as mentioned in abstract) and revise results section.

 Discussion: This sections can be built on key findings. At present, it looks too generic.

 Conclusions need to re-write. It should be authors' learning based on results.

 Conclusion section doesn't require citation.  Remove the sentence, "It is hoped that understanding these barriers will inform future interventions for anaemia in Kano and help to improve services."

 "Many of these findings may be generalizable to other parts of Nigeria when providing care for maternal anaemia. " On what basis authors claim the generalization of findings? Such statements should be avoided

Is the work clearly and accurately presented and does it cite the current literature?

Partly

If applicable, is the statistical analysis and its interpretation appropriate?

Yes

Are all the source data underlying the results available to ensure full reproducibility?

Yes

Is the study design appropriate and is the work technically sound?

Yes

Are the conclusions drawn adequately supported by the results?

Partly

Are sufficient details of methods and analysis provided to allow replication by others?

Partly

Reviewer Expertise:

Public health, qualitative research, maternal health, health technology assessment

I confirm that I have read this submission and believe that I have an appropriate level of expertise to confirm that it is of an acceptable scientific standard, however I have significant reservations, as outlined above.

# Response

**Published:** 2023-10-29

**Authors:** Tara Tancred

**Affiliations:** Liverpool School of Tropical Medicine, UK

Many thanks to the reviewer for their thoughtful reading of our manuscript. We have indicated our responses inline below in bold:

- As majority participants are from Hausa or Fulani ethnic group, there should be brief paragraph on ethic groups in Nigeria.

***We have added slightly more on this.***

 Methodology: 

 Authors should re-organize this section as follows:

- Research design: Specify which qualitative research design was used.

***We note that this was a qualitative study but have added “phenomenological”.***

- Study setting: provide justification why this setting was selected.

***This is evident from the introduction and indication that Kano state has the highest levels of maternal anaemia in Nigeria. We have further noted that the hospital in particular draws a wide population, making it a useful site to study from a diverse range of participants.***

- Explain some characteristics of this ethic group. Hausa or Fulani ethnic groups 

***We have noted that they are Muslim. But feel that most of the cultural implications of collecting data from this population are expressed in the qualitative findings.***

- Sampling technique should be explicitly mentioned.

***This is explicit under “Sampling and data collection”. All participants were sampled purposively.***

- Research tools: Explain how each research tools were developed.

***This is also explicit under “Sampling and data collection”. We describe the contents of the instruments and how we arrived at those.***

- Data collection procedure: Explain who and how data was collected? In which language data was collected? Was data collector trained in qualitative interviews/qualitative research? What was the duration of data collection? 

***This is also explicitly stated: “All interviews and FGDs were carried out in Hausa or English. All interviews and FGDs were audio-recorded, transcribed verbatim, and later translated into English if necessary. Handwritten notes to capture non-verbal communication were made during interviews and FGDs by a research assistant. FGDs took 45–70 minutes.” We added in a point about the length of data collection for interviews and noted that AKG carried out the data collection and that she has extensive training in social science methodology.***

 Ethical considerations: Explain how authors ensured human rights protection of participants. Explain how authors dealt with ethical dilemma if they had encountered.

***No ethical dilemmas were encountered. We otherwise are explicit in our upholding of informed consent, privacy, and confidentiality. This is detailed already under “Ethics and consent”.***

 How qualitative data managed need to be explained (deidentification, storage of data, protection of privacy, etc.)

***It is clear from our preceding text how data were de-identified (we note that all data were anonymised). We have added in slightly more detail around providing a unique identifier and encrypting digital files.***

 Data analysis: Analytic framework used by authors should be explained with appropriate reference (citation) and steps followed in analyzing qualitative data.

***We have explicitly noted that “thematic analysis” was used, though the steps were already clear. There is no analytic framework.***

 Results: This section is poor. It looks superficial. Authors claim inductive coding was used; however, results do not reflect that.  It lacks triangulation of data from different sources and analytic rigour. Authors should follow thematic analytic framework (as mentioned in abstract) and revise results section.

***Thank you for the feedback, but we respectfully disagree. It is very clear from participants that the overarching themes were reiterated. There is obvious triangulation in that you can clearly see supporting content from different types of participants. These perhaps come across as superficial because they are very “top line” as they encompass many things. We synthesised data into the most top line themes, and this is what emerged. We have made this more of a narrative statement that better encompasses the contents within each theme.***

 Discussion: This sections can be built on key findings. At present, it looks too generic.

***We built our discussion on key findings—we reflect on these against broader literature, as would be expected within a discussion.***

 Conclusions need to re-write. It should be authors' learning based on results.

 Conclusion section doesn't require citation.  Remove the sentence, "It is hoped that understanding these barriers will inform future interventions for anaemia in Kano and help to improve services."

***We have slightly reorganised the conclusion, moving the cited text to earlier in the discussion and rephrasing the identified sentence.***

 "Many of these findings may be generalizable to other parts of Nigeria when providing care for maternal anaemia. " On what basis authors claim the generalization of findings? Such statements should be avoided

***We have reflected throughout our discussion about the persistent constraints in anaemia care in other parts of Nigeria and how resonant our findings were, hence this statement.***
